# The Suppression of miR-199a-3p by Promoter Methylation Contributes to Papillary Thyroid Carcinoma Aggressiveness by Targeting RAP2a and DNMT3a

**DOI:** 10.3389/fcell.2020.594528

**Published:** 2020-12-07

**Authors:** Feng Wu, Xiao Lin, Su-Kang Shan, Fuxingzi Li, Feng Xu, Jia-Yu Zhong, Bei Guo, Ming-Hui Zheng, Yi Wang, Zhao-Hui Mo, Ling-Qing Yuan

**Affiliations:** ^1^Department of Pathology, The Second Xiangya Hospital, Central South University, Changsha, China; ^2^Department of Metabolism and Endocrinology, National Clinical Research Center for Metabolic Diseases, The Second Xiangya Hospital, Central South University, Changsha, China; ^3^Department of Radiology, The Second Xiangya Hospital, Central South University, Changsha, China; ^4^Department of Endocrinology, The Third Xiangya Hospital, Central South University, Changsha, China

**Keywords:** papillary thyroid cancer, microRNA, DNA methylation, DNMT, Rap2a

## Abstract

**Background:**

It was previously demonstrated that miR-199a-3p plays an important role in tumor progression; especially, its down-regulation in papillary thyroid cancer (PTC) is associated with cancer cell invasion and proliferation. In the present report, we investigated the mechanism involved in the down-regulation of miR-199a-3p in PTC and how miR-199a-3p regulates PTC invasion both *in vivo* and *in vitro*.

**Methods:**

qRT-PCR and Western blot assays were used to determine the expression of the investigated genes. Bisulfite sequencing PCR was used to investigate miR-199a-3p methylation. The functions of miR-199a-3p were investigated by a series of *in vitro* and *in vivo* experiments.

**Results:**

Our results showed hypermethylation of the miR-199a-3p promoter, which resulted in decreased miR-199a-3p expression both in PTC cell lines and PTC tissues. DNA-methyltransferase 3a (DNMT3a), a target gene of miR-199a-3p, was increased both in PTC cell lines and PTC tissues, while 5-aza-2′-deoxycytidine (methyltransferase-specific inhibitor) or knock-down using DNMT3a Small-Interfering RNA could restore the expression of miR-199a-3p, and the over-expression of miR-199a-3p could decrease the expression of DNMT3a; this suggests that miR-199a-3p/DNMT3a constructs a regulatory circuit in regulating miR-199a-3p/DNMT3a expression. Moreover, gain- and loss-of-function studies revealed that miR-199a-3p is involved in cancer cell migration, invasion, and growth. Meanwhile, we found that RAP2a was also a direct target of miR-199a-3p, which might mediate the tumor-growth-inhibiting effect of miR-199a-3p. To further confirm the tumor-suppressive properties of miR-199a-3p, stable overexpression of miR-199a-3p in a PTC cell line (BCPAP cells) was xenografted to athymic BALB/c nude mice, resulting in delayed tumor growth *in vivo*. In clinical PTC samples, the expression of RAP2a and DNMT3a was increased significantly, and the expression of RAP2a was inversely correlated with that of miR-199a-3p.

**Conclusion:**

Our studies demonstrate that an epigenetic change in the promoter region of miR-199a contributes to the aggressive behavior of PTC via the miR-199a-3p/DNMT3a regulatory circuit and directly targets RAP2a.

## Introduction

DNA methylation, which involves the translocation of a methyl group to the number 5 carbon of the cytosine ring in a CpG dinucleotide, plays a crucial role in regulating gene expression in cancer development (Raynal et al., 2012). In cancer cells, transcriptional modulation of tumor suppressor genes (TSGs) or oncogenes by DNA methylation of promoter-associated CpG islands is a hallmark of carcinogenesis (Fernandez et al., 2012). DNA methylation is mediated by several DNA-methyltransferases (DNMT), including DNMT1, DNMT3a, and DNMT3b (Jeltsch and Jurkowska, 2016). DNMT3a and DNMT3b play a role in *de novo* methylation, while DNMT1 is critical for the maintenance of methylation (Liao et al., 2015). Abnormal DNMT expression will result in the alteration of gene expression.

Thyroid cancer is the most common endocrine malignancy, and its incidence has increased over the past few decades (Jemal et al., 2008). The most prevalent histological subtype of thyroid cancer is papillary thyroid carcinoma, which accounts for over 80% of all thyroid cancer cases (Aschebrook-Kilfoy et al., 2013). Most PTC patients can be treated successfully by surgical resection with radioactive iodine and thyroid hormone administration. However, approximately 10–20% of patients present with recurrences and distant metastases (Randle et al., 2017). The mechanisms that regulate tumor initiation and progression have not been fully elucidated. It has been reported that miR-199a-3p plays tumor suppressor functions in the carcinogenesis of PTC (Liu et al., 2017), and miR-199a-3p was generally hypermethylated in malignant testicular tumors (Cheung et al., 2011; Gu et al., 2013; Chen et al., 2014) and ovarian cancer (Deng et al., 2017), which correlated with its down-regulation. However, the mechanism by which miR-199a is down-regulated in PTC and functions as a TSG has not been fully elucidated. Therefore, we hypothesize that aberrant DNA methylation in miR-199a is a factor in the development of PTC.

In this study, we document the general hypermethylation of miR-199a in PTC, which correlates with its down-regulation. The lower expression of miR-199a-3p resulted in an increase in PTC cell invasion and migration, while the increased expression of DNMT3a may explain the hypermethylation of miR-199a in PTC tissues and cells. Moreover, we identified DNMT3a and RAP2a as target genes of miR-199a-3p. Furthermore, 5-aza-2′-deoxycytidine (a methyltransferase-specific inhibitor) or knock-down using DNMT3a Small-Interfering RNA (siRNA) could restore the expression of miR-199a-3p, and the overexpression of miR-199a-3p could decrease the expression of DNMT3a, which suggested that the miR-199a-3p/DNMT3a construct was part of a regulatory circuit controlling miR-199a-3p/DNMT3a expression. RAP2a is a novel target of p53 and is induced upon DNA damage in a p53-dependent manner (Wu et al., 2015). RAP2a is significantly up-regulated in many types of tumors; the ectopic expression of RAP2a plays a key role in enhancing the migration and invasion ability of cancer cells (Prabakaran et al., 2011; Lee et al., 2015; Wu et al., 2015). We found that RAP2a expression was aberrantly up-regulated in PTC and inversely correlated with miR-199a-3p expression. The depletion of RAP2a suppressed cancer invasion and migration. In clinical PTC samples, the expression of RAP2a and DNMT3a was significantly increased, and the expression of RAP2a was inversely correlated with that of miR-199a-3p compared with the control. Our data imply that an epigenetic change in the promoter region of miR-199a contributes to the aggressive behavior of PTC via a regulatory circuit involving miR-199a-3p/DNMT3a and targets RAP2a directly.

## Materials and Methods

### Ethics Statement

All animal and human studies were carried out under the approval and supervision of the ethics committee of the Second Xiangya Hospital, Central South University. The human study and human samples conformed to the principles outlined in the Declaration of Helsinki. Written informed consent was obtained from all participants in our experiments.

### Patients and PTC Tissue Samples

A total of 60 pairs of thyroid tissues from PTC patients with lymph node metastasis and donors were obtained from the Department of Breast and Thyroid, the Second Xiangya Hospital of Central South University. The clinical characteristics of all patients and donors are shown in the Supplementary Table 1. The donors were diagnosed with benign thyroid nodule, and the PTC was diagnosed according to the WHO classification evaluated by two pathologists, and representative tumor areas and normal thyroid tissue were selected.

### Cell Culture

Two human papillary thyroid carcinoma cell lines, BCPAP and KTC-1, were obtained from the Chinese Academy of Sciences (Shanghai, China). The cells were cultured in RPMI 1640 medium (Gibco BRL; Grand Island, United States) supplemented with 10% fetal bovine serum (FBS; Gibco BRL; Grand Island, United States), 1% penicillin/streptomycin (PS; Gibco BRL; Grand Island, United States), 1% non-essential amino acids (NEAA; Invitrogen 11140050), 1% Glutamax (Invitrogen 35050061), and 1% sodium pyruvate solution (Invitrogen 11360070). The human normal thyroid cell line (Nthy-ori-3-1; CellBio, Shanghai, China) was cultured in high-glucose DMEM (Sigma; St Louis, MO, United States) containing 10% FBS and 1% PS. Cells were maintained at 37°C with 5% CO_2_ in a humidified environment.

### Isolation of DNA From Archived Tissues and Cultured Cells

Total genomic DNA was isolated from papillary thyroid carcinoma tissue samples, normal thyroid tissue samples, BCPAP cells, KTC-1 cells, and Nthy-ori-3-1 cells, by using a QIAamp DNA mini kit (Qiagen, Germany). DNA was digested and purified using an EpiTect Bisulfite kit (Qiagen, Germany). All procedures were performed according to the manufacturer’s instructions.

### Methylation Analysis Using Bisulfite Genomic Sequencing PCR

Bisulfite sequencing PCR (BSP) was conducted as described previously (Lin et al., 2018). Bisulfite-treated human miR-199a containing 13 CpG sites on chromosome 1 and 33 CpG sites on chromosome 19 was amplified with the primers 199a-1-upF1 (5′-GGATATGAGATTTAAAAAAGGAG-3′) and 199a-1-upR3 (5′-CAAACAAACAAACAAACAAAAAC-3′), the primers 199a-19-upF1 (GTGTTTTTTTTTTTATTTAG) and 199a-19-upR2 (RAAAACTTCCAACCAACAAA). The amplified PCR products were purified and subcloned into the pGEM-T Easy vector (Promega, Madison, WI, United States). In total, 10 clones of experimental samples were sequenced. The percentage of methylated CpG dinucleotides was calculated to evaluate the methylation level of miR-199a-3p.

### Small-Interfering RNA and miRNA Transfection

Transfections with siRNA and miRNA were performed as previously described (Peng et al., 2017). BCPAP and KTC-1 cells were seeded into a six-well plate. For the transient transfection of miR-199a-3p mimics, mimic controls, miR-199a-3p inhibitors, inhibitor controls, RAP2a siRNA oligonucleotides or scrambled siRNA controls (Gene Pharma; Shanghai, China), a combination of oligonucleotides (20 nM) and 10 μl Lipofectamine 2000 (Invitrogen; Carlsbad, CA, United States) were mixed following the manufacturer’s instructions. After 6 h of co-culture, the cells were placed in complete medium for 24 to 48 h before further assay.

### Transwell Migration and Invasion Assays

Transwell migration and invasion assays were performed as described previously (Xu et al., 2020). Briefly, before cell seeding, the upper Transwell chambers (8 μm pore size; Corning Inc., Union City, CA, United States) were either coated with a Matrigel matrix or left uncoated (BD Biosciences, San Diego, CA, United States). Then, 5 × 10^4^ transfected BCPAP or KTC-1 cells suspended in 200 μl of serum-free RMPI 1640 were added to the top of each well insert. A medium with 20% FBS was placed in the bottom wells. The cells were then allowed to migrate for 48 h at 37°C. Invasive cells, those that migrated to the lower surface of the membrane and bottom of the plate, were fixed with 10% methanol for 15 min and then stained with 1% crystal violet for 10 min. The stained cells were counted under a light microscope (Olympus, Japan). To minimize bias, at least five fields were counted under 100× magnification, and the various counts were averaged.

### Colony Formation Assay

BCPAP or KTC-1 cells were transfected with miR-199a-3p mimics, mimic controls, siRAP2a, or negative control siRNA and then grown in six-well plates for 24 h. For each group, 1,000 cells were seeded in triplicate into six-well plates for 7 days, during which period the medium was not changed. Cells were then fixed with 10% methanol, stained with 1% crystal violet for 5 min and washed three times with PBS.

### Plasmid Constructs and Luciferase Reporter Assay

To analyze the function of miR-199a-3p, we cloned segments of the RAP2a and DNMT3a 3′-UTR, including the predicted miR-199a-3p binding sites, into the *Pme*I and *Xba*I restriction sites of the luciferase reporter vector pmirGLO (Promega, Madison, Wisconsin), producing a wild-type RAP2a 3′-UTR (WT-RAP2a-3′-UTR) and DNMT3a 3′-UTR (WT-DNMT3a-3′-UTR). RAP2a and DNMT3a mutants of the miR-199a-3p seed regions were produced using the QuikChange Site-Directed Mutagenesis kit (Stratagene, United States) to obtain a mutant RAP2a 3′-UTR (MUT-RAP2a-3′-UTR) and a mutant DNMT3a 3′-UTR (MUT-DNMT3a-3′-UTR).

BCPAP cells were co-transfected with a luciferase reporter carrying WT-RAP2a-3′-UTR, MUT-RAP2a-3′-UTR, WT-DNMT3a-3′-UTR, or MUT-DNMT3a-3′-UTR, together with miR-199a-3p mimic or mimic control. 48 h after transfection, luciferase activities were detected using the luciferase assay system (Promega Corp. United States). The sequences of the PCR and mutagenic primers are shown in the Supplementary Table 2.

### Quantitative Real-Time RT-PCR

Total RNA was extracted from cultured cells, patient sera, or human thyroid tissues by using TRIzol reagent (Invitrogen). For human thyroid tissues, the tissue was first cut into small fragments with tissue scissors, and then the TRIzol reagent was added. For the detection of mRNA, cDNA was synthesized from 1 μg of total RNA using an All-in-One^TM^ first-strand cDNA synthesis kit (Genecopoeia; Guangzhou, China). Then, a 25-μl reverse-transcription reaction was carried out for 10 min at 65°C, 60 min at 37°C, 5 min at 85°C, and a final hold at 4°C. Quantitative PCR (qPCR) analysis was performed with All-in-One^TM^ qPCR Mix (Genecopoeia; Guangzhou, China) in a LightCycler^®^ 96 System (Roche, Indianapolis, IN, United States). For qPCR analysis, 20-μl reactions were incubated in a 96-well optical plate at 95°C for 10 min, followed by 40 cycles of 95°C for 10 s, 62°C for 20 s, and 72°C for 15 s. Data were normalized to β-actin values. The following PCR primers used in this study were purchased from Genecopoeia: DNMT1, DNMT3a, DNMT3b, rap2a, β-actin, miR-199a-3p, miR-199a-5p, and U6 small nuclear RNA. The relative standard curve method was used to determine relative mRNA and miRNA expression. Results were expressed as fold changes relative to the corresponding controls. The qPCRs were performed in triplicate; results are presented as the mean ± SD of samples.

### Western Blot Analysis

Western blotting was carried out as described previously (Lin et al., 2019; Wu et al., 2019). Briefly, the proteins from each cell layer extract were quantified using the BCA Protein kit (Beyotime Biotechnology, Shanghai, China). Protein extracts were separated by SDS-PAGE and transferred to polyvinylidene fluoride membranes (Millipore, Billerica, MA, United States). Membranes were blocked with 5% non-fat milk in Tris–buffered saline for 1 h at room temperature. The membranes were incubated with primary antibodies, including anti-RAP2a (1:2,000; catalog no. NBP2-24574; Novusbio), DNMT3a (1:500; catalog no. 2160; Cell Signaling Technology), and anti-β-actin (1:3,000; catalog no. AP53385; Abgent) at 4°C overnight, followed by incubation with HRP-conjugated goat anti-rabbit (1:5,000; catalog no. sc-2004; Santa Cruz Biotechnology; RRID:AB_631746) or HRP-conjugated goat anti-mouse (1:5,000; sc-2005; Santa Cruz Biotechnology; RRID:AB_631746) secondary antibodies at 37°C for 1 h. The immunoreactive bands were visualized by using the ECL Plus Western Blot Detection kit (Amersham Biosciences United Kingdom), and densitometric quantification of band intensity from three independent experiments was carried out with the Image-Pro Plus 6.0 software. The relative expression level of the target protein was normalized to the intensity of the β-actin band.

### Establishment of Stable Cell Lines and Animal Experiments

To establish the stable cell lines BCPAP-NC and BCPAP-199a-3p, BCPAP cells were infected with LV16-NC or LV16-miR-199a-3p (Gene Pharma; Shanghai, China). Selection was initiated 24 h post-transfection in the BCPAP cell line with 0.25 mg/ml puromycin. Cell suspensions of 1 × 10^6^ cells in 100 μl of PBS were used for the subcutaneous injection of 4-week-old male athymic BALB/c nude mice. Each group contained five mice. 8 weeks later, D-luciferin was administered to each mouse by intraperitoneal injection at a dose of 150 mg/kg, and the mice were anesthetized for 5 min in a chamber with 3% isoflurane. The mice were then imaged by using a Xenogen IVIS Lumina II imaging system.

### Immunohistochemical Staining

Immunohistochemical staining for rap2a was performed as previously described (Wang et al., 2017). Rap2a expression was assayed on papillary thyroid cancer (PTC) tissues (*n* = 5) and on histologically normal thyroids (*n* = 5). Slides were counterstained with hematoxylin, and images were taken with a light microscope (Olympus).

### Statistical Analysis

Experimental results are presented as means ± SD, and analysis was performed with Statistical Product and Service Solutions (SPSS; Chicago, IL, United States) software (version 19.0). Comparisons of values between more than two groups were evaluated by analysis of variance (one-way ANOVA). The correlations between miR-199a-3p expression and RAP2a level were analyzed by Spearman’s rank correlation. A level of *p* < 0.05 was considered statistically significant. All experiments were repeated at least three times, and representative experiments are shown in the figures.

## Results

### The miR-199a Promoter Is Hypermethylated in PTC, and miR-199a-3p Is Significantly Down-Regulated in PTC Compared With Normal Thyroid Tissue and Cells

**FIGURE 1:**
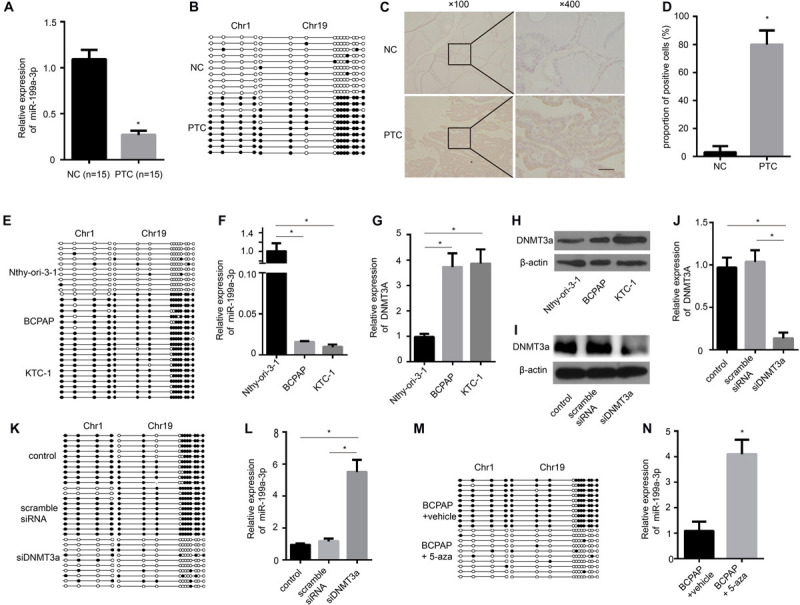
The miR-199a promoter is hypermethylated in papillary thyroid cancer (PTC), and miR-199a-3p is significantly down-regulated in PTC compared with normal thyroid tissue and cells. **(A)** qRT-PCR showing the expression of miR-199a-3p in non-cancerous thyroid tissues and PTC tissues. *n* = 15. **(B)** A schematic illustration of the location of CpG islands upstream of miR-199a-3p DNA isolated from non-cancerous thyroid tissues or PTC tissues. **(C)** The expression of DNMT3a was assayed by immunohistochemistry staining. **(D)** A proportion of the positive cells in **(C)** were analyzed. **(E)** A schematic illustration of the location of CpG islands upstream of miR-199a-3p DNA isolated from Nthy-ori-3-1, BCPAP, or KTC-1 cell lines. **(F)** qRT-PCR showing the expression of miR-199a-3p in Nthy-ori-3-1, BCPAP, or KTC-1 cell lines. **(G,H)** The expression of DNMT3a was assayed by qRT-PCR and Western blot analysis in Nthy-ori-3-1, BCPAP, or KTC-1 cell lines. **(I)** Western blot analysis showed that DNMT3a was knocked down successfully by DNMT3a siRNA. **(J)** qRT-PCR showing that the expression of DNMT3a was knocked down successfully by DNMT3a siRNA. **(K)** A schematic illustration of the location of CpG islands upstream of miR-199a-3p DNA isolated from KTC-1 cells treated with or without siDNMT3a. **(L)** qRT-PCR showing the expression of miR-199a-3p in KTC cells with DNMT3a knocked down. **(M)** A schematic illustration of the location of CpG islands upstream of miR-199a-3p DNA isolated from KTC-1 cells treated with or without 5-aza (10 μmol/L). **(N)** qRT-PCR showing the expression of miR-199a-3p in KTC cells treated with or without 5-aza. Data are presented as mean ± S.D. from triplicate experiments. *<0.05. Bars = 200 μm.

To determine whether the levels of miR-199a-3p are down-regulated in clinical PTC samples, we used quantitative RT-PCR (qRT-PCR) to analyze RNAs from 15 paired normal thyroid tissues and PTC tissues. We found significantly lower expression of miR-199a-3p in PTC tissues compared with normal thyroid tissues (Figure 1A). These results suggested that miR-199a-3p might be a TSG. Then, we investigated the mechanism involved in miR-199a-3p down-regulation in PTC. The increased methylation of promoters is one mechanism of transcriptional silencing. We then performed bisulfite sequencing on PTC tissues and normal thyroid tissues. Our results showed that the promoter of the miR-199a-3p locus in PTC tissues was highly methylated, whereas it was hypomethylated in normal thyroid tissues (Figure 1B). DNA methylation is regulated by DNMTs, including DNMT1, DNMT3a, and DNMT3b. To investigate this regulatory mechanism involving different methylation states of the miR-199a promoter, we analyzed the expression of DNMTs in PTC and normal thyroid tissue. We found an obvious increase in the expression of DNMT3a by IHC in PTC tissues in comparison with normal thyroid tissues (Figures 1C,D). However, neither DNMT1 nor DNMT3b expression was significantly different between PTC tissues and normal thyroid tissues (Supplementary Figures 1A,B). Therefore, we performed bisulfite sequencing on two PTC cell lines, BCPAP and KTC-1, and the normal thyroid follicular cell line Nthy-ori-3-1. The promoter of the miR-199a locus in both cancer lines was highly methylated, whereas it was hypomethylated in the non-cancerous thyroid cell line (Figure 1E). To further confirm that aberrant methylation of miR-199a is related to miR-199a-3p expression, we measured its expression by qRT-PCR. In comparison with Nthy-ori-3-1, the expression of miR-199a-3p was significantly down-regulated in PTC cell lines (Figure 1F). We also observed a significant up-regulation of DNMT3a in BCPAP cells and KTC-1 cells when compared with normal Nthy-ori-3-1 thyroid follicular cells (Figures 1G,H). To explore whether DNMT3a plays a central role in regulating the expression of miR-199a-3p, we transfected DNMT3a siRNA into BCPAP cells to knock down the expression of DNMT3a. The inhibitory efficiency of siDNMT3a was verified by Western blotting (Figure 1I) and qRT-PCR (Figure 1J). The transfection of BCPAP cells with siDNMT3a remarkably decreased the methylation level of BCPAP cells (Figure 1K). Then, we found that knocking down the expression of DNMT3a significantly down-regulated the expression of miR-199a-3p (Figure 1L). Moreover, the treatment of BCPAP cells with 5-aza-2′-deoxycytidine (methyltransferase-specific inhibitor) remarkably decreased the methylation level of BCPAP cells (Figure 1M) and increased the expression level of miR-199a-3p (Figure 1N). These results indicate that increasing the expression of DNMT3a leads to the hypermethylation of miR-199a-3p, which results in the decreased expression of miR-199a-3p in PTC tissues and cell lines.

### Expression of miR-199a-3p Suppresses Cancer Migration, Invasion, and Cell Growth *in vitro*

**FIGURE 2:**
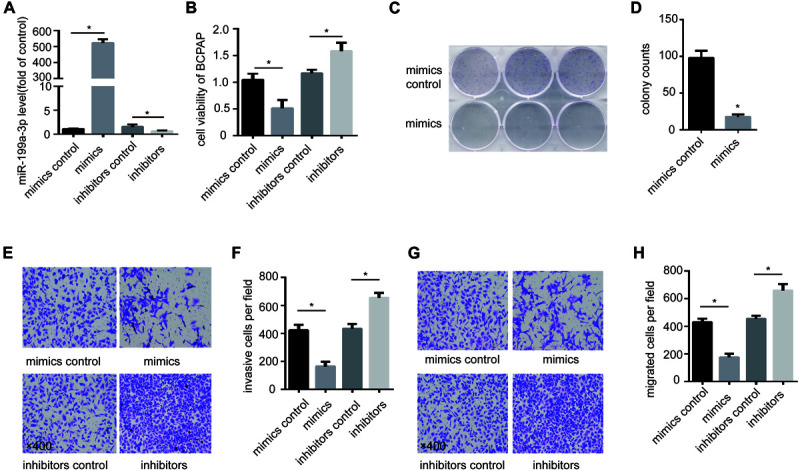
Expression of miR-199a-3p suppresses cancer cell migration, invasion and growth *in vitro*. **(A)** BCPAP cells were transfected with miR-199a-3p mimics, mimic controls, miR-199a-3p inhibitors or inhibitor controls, and subjected to qRT-PCR analysis of miR-199a-3p. **(B)** BCPAP cells were transfected with miR-199a-3p mimics, mimic controls, miR-199a-3p inhibitors or inhibitor controls, and subjected to a cell viability assay. **(C)** BCPAP cells were transfected with either miR-199a-3p mimics or mimic controls for 24 h, and 1,000 cells were seeded into six-well plates for 7 days to assay colony formation ability. **(D)** The colonies were counted, and the results were presented as mean ± S.D. from three individual experiments. **(E)** miR-199a-3p mimics inhibited, while miR-199a-3p inhibitors increased invasion ability of BCPAP. **(F)** The number of invasive cells was counted, and the results were presented as mean ± S.D. from three individual experiments. **(G)** miR-199a-3p mimics inhibited, while miR-199a-3p inhibitors increased the migration ability of BCPAP. **(H)** The number of migrated cells was counted, and the results were presented as mean ± S.D. from three individual experiments. *p < 0.05. Bars = 200 μm.

To study the function of miR-199a-3p in PTC, we overexpressed miR-199a-3p in BCPAP cells with miR-199a-3p mimics and inhibit miR-199a-3p in BCPAP cells with miR-199a-3p inhibitors (Figure 2A). We found that miR-199a-3p mimics markedly attenuated BCPAP cell proliferation compared with the control group through a CCK-8 proliferation assay (Figure 2B) and a colony formation assay (Figures 2C,D). We also found that miR-199a-3p mimics inhibited invasion (Figures 2E,F) and migration (Figures 2G,H), while miR-199a-3p inhibitors stimulated invasion (Figures 2E,F) and migration (Figures 2G,H) in BCPAP cells compared with the control group. These observations suggest that miR-199a-3p can suppress the proliferation, migration, and invasion of PTC cells *in vitro*.

### DNMT3a and RAP2a Are Direct Targets of miR-199a-3p

**FIGURE 3:**
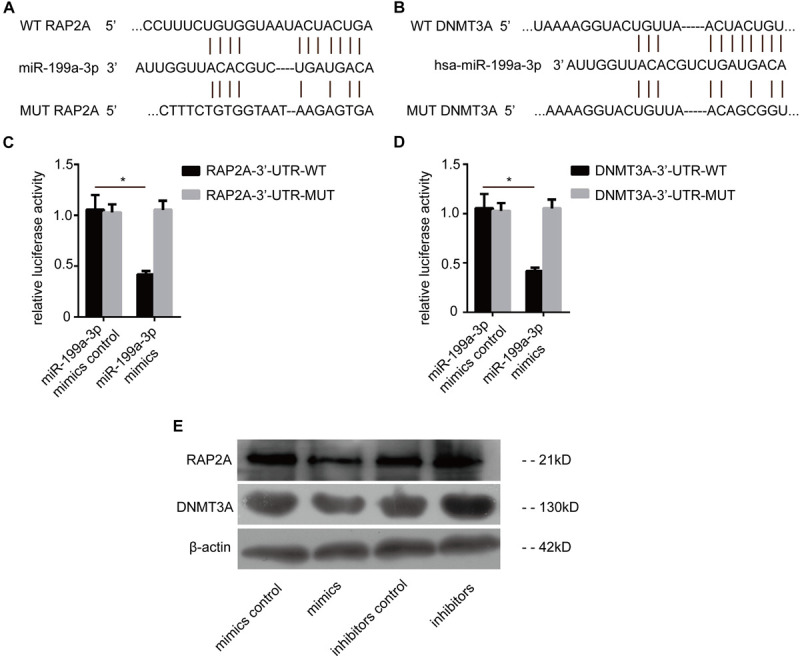
RAP2a and DNMT3a are the direct targets of miR-199a-3p. **(A)** Schematic of the putative miR-199a-3p target site in the human RAP2a 3′-UTR. **(B)** Schematic of the putative miR-199a-3p target site in the human DNMT3a 3′-UTR. **(C)** KTC-1 cells were co-transfected with the luciferase reporter carrying the WT-RAP2a-3′-UTR or MUT- RAP2a -3′-UTR, and miR-199a-3p or control miR mimics. Firefly luciferase values obtained 48 h after transfection and normalized to Renilla luciferase are presented. **(D)** BCPAP cells were co-transfected with the luciferase reporter carrying WT-DNMT3a-3′-UTR or MUT- DNMT3a-3′-UTR, and miR-199a-3p or control miR mimics. Firefly luciferase values obtained 48 h after transfection and normalized to Renilla luciferase are presented. **(E)** RAP2a and DNMT3a protein levels obtained by Western blot analysis (30 μg of protein was used for each Western blot performed) were tested 2 days after BCPAP cells were transfected with miR-199a-3p mimics, mimic controls, miR-199a-3p inhibitors, or inhibitor controls. *p < 0.05.

To explore the downstream mechanism of miR-199a-3p in PTC cells, the computational algorithm TargetScan was used to search for potential miR-199a-3p target genes. Interestingly, the results showed that miR-199a-3p was predicted to have a potential miRNA binding site in the 3′-UTR of RAP2a mRNA and DNMT3a mRNA (Figures 3A,B). To confirm that RAP2a and DNMT3a are direct targets of miR-199a-3p, we conducted a reporter assay using the luciferase reporter plasmid containing the wild type (WT) and mutated (MT) RAP2a 3′-UTR with the miR-199a-3p binding site. Transfection with miR-199a-3p mimics inhibited the luciferase activity of the WT RAP2a 3′-UTR, but the inhibitory ability of mutant RAP2a 3′-UTR vectors was compromised (Figure 3C). We also conducted a reporter assay using the luciferase reporter plasmid containing the WT and MT DNMT3a 3′-UTR with the miR-199a-3p binding site. Transfection with miR-199a-3p mimics inhibited the luciferase activity of the WT DNMT3a 3′-UTR, but the inhibitory ability was compromised for mutant DNMT3a 3′-UTR vectors (Figure 3D). We transfected miR-199a-3p mimics into BCPAP cells and found that miR-199a-3p mimics reduced RAP2a and DNMT3a protein levels (Figure 3E), while the miR-199a-3p inhibitor increased its protein levels. These results suggest that miR-199a-3p directly recognizes the 3′-UTR of RAP2a and DNMT3a, and thereby inhibits their translation.

### RAP2a Promotes Cancer Cell Migration, Invasion, and Growth

**FIGURE 4:**
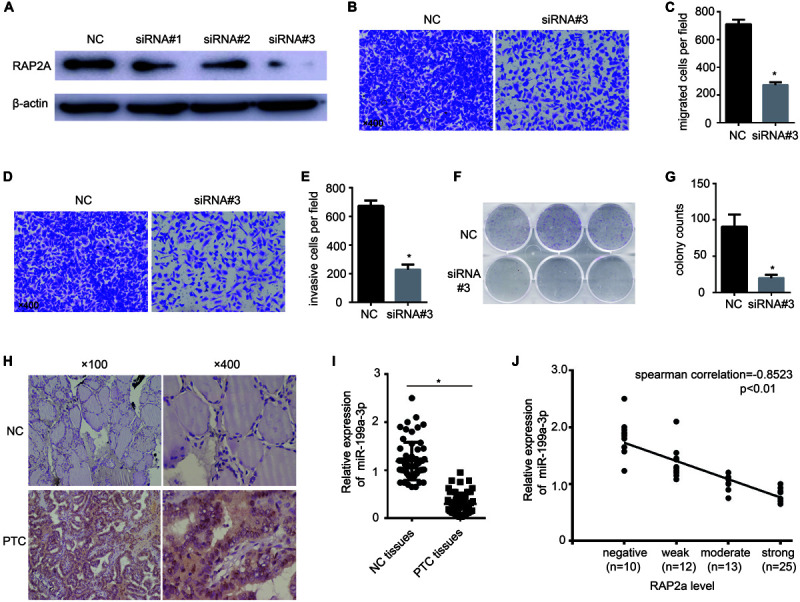
Rap2a promotes cancer cell migration, invasion, and growth. **(A)** The inhibitory efficiency of siRNAs targeting RAP2a was verified by western blotting. **(B)** Migration of BCPAP cells was tested by using the Transwell migration assay. **(C)** Quantitative analysis of the migrated cells in **(B)**. *n* = 3 per group. **(D)** The invasiveness of BCPAP cells was tested by using the Transwell invasion assay. **(E)** Quantitative analysis of the invasive cells in **(D)**. *n* = 3 per group. **(F)** BCPAP cells were transfected with either negative control or siRAP2a for 24 h, and 1,000 cells were seeded into six-well plates for 7 days to assay colony-formation ability. **(G)** The colonies were counted, and the results are presented as mean ± S.D. from three individual experiments. **(H)** The expression of RAP2a in non-cancerous thyroid tissues and PTC tissues was assayed by immunohistochemistry staining. **(I)** qRT-PCR showing the expression of miR-199a-3p in non-cancerous thyroid tissues and PTC tissues. *n* = 60. **(J)** Scatter plots of miR-199a-3p expression against the RAP2a level. *p < 0.05. Bars = 200 μm.

To further confirm that RAP2a plays a central role in regulating cancer progression, we transfected siRAP2a into BCPAP cells to knock down the expression of RAP2a. Of the three independent small interfering RAP2a sequences we designed, Western blot analysis showed that only the third siRAP2a could successfully knock down the expression of RAP2a (Figure 4A). Therefore, we chose the third siRAP2a for further study. We found that siRAP2a transfection inhibited the *in vitro* migration potential of BCPAP cells compared with the control group (Figures 4B,C). Moreover, siRAP2a transfection inhibited the invasive potential of BCPAP cells compared with the control group *in vitro* (Figures 4D,E). We also noticed that siRAP2a transfection markedly attenuated BCPAP cell proliferation compared with the control group through a colony formation assay (Figures 4F,G). Furthermore, we examined RAP2a protein expression levels (Figure 4H) and the relationship between RAP2a expression and miR-199a-3p expression levels (Figure 4I) by analyzing PTC tissue specimens, as well as normal thyroid tissues. The strong positive expression of RAP2a was identified in PTC tissues, but only weak staining was observed in normal thyroid tissues (Figure 4H). In contrast, miR-199a-3p was identified as highly expressed in normal thyroid tissues, and much lower expression was detected in PTC tissues (Figure 4I). When the samples were grouped according to RAP2a intensity, in these 60 patients’ specimens, we found that the Spearman’s rank correlation test showed a negative correlation between the RAP2a level and miR-199a-3p (Figure 4J). These results indicated that miR-199a-3p has tumor suppressor functions, and that RAP2a might be a tumor-promoting gene in PTC.

### MiR-199a-3p Suppresses Cancer Development in a Mouse Xenograft Model

**FIGURE 5:**
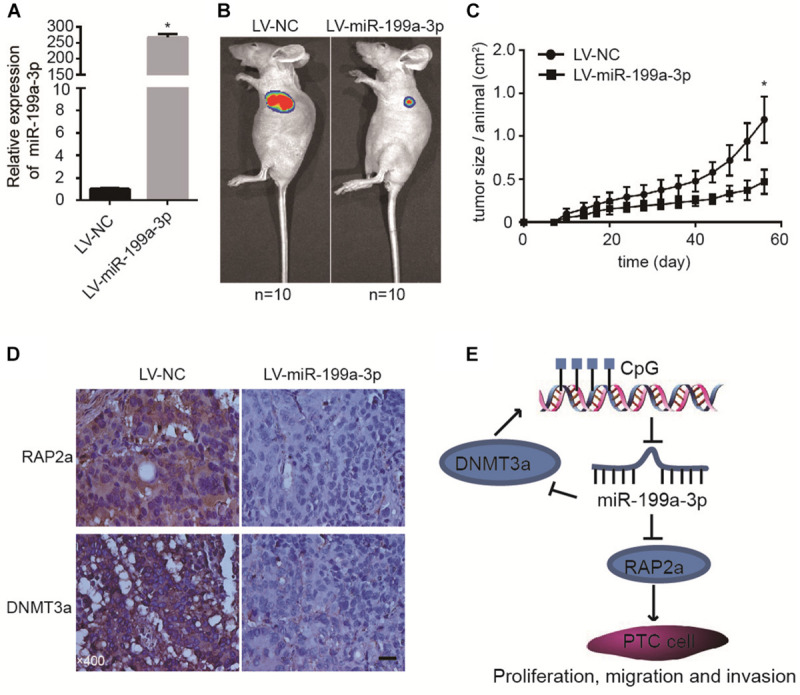
MiR-199a-3p suppresses cancer development in a mouse xenograft model. **(A)** BCPAP cells were infected with LV-NC or LV-miR-199a-3p and subjected to qRT-PCR analysis of miR-199a-3p. **(B)** Representative images from IVIS imaging. **(C)** Tumor volume assessment by digital caliper every 4 days shows that tumor growth in the LV-miR-199a-3p group is much slower than that in the control group. **(D)** The expression of RAP2a and DNMT3a was assayed by immunohistochemistry staining of mouse xenograft tissues. **(E)** A proposed model of the miR-199a-3p/DNMT3a/RAP2a regulatory circuit involved in the proliferation, migration, and invasion of PTC. *p < 0.05. Bars = 200 μm.

To further confirm the tumor suppressive properties of miR-199a-3p, we used a xenograft animal model to study its function *in vivo*. We induced the constitutive expression of miR-199a-3p and luciferase in cancer cells with lentivirus. Cells expressing miR-199a-3p were selected by puromycin. These cells (BCPAP-LV-miR-199a-3p) demonstrated a greater than 200-fold increase in miR-199a-3p expression (Figure 5A) when compared with vector-infected control cells (BCPAP-LV-NC). Then, equal numbers of BCPAP-LV-miR-199a-3p and BCPAP-LV-NC cells were subcutaneously injected into the left flank of athymic nude mice (*n* = 10 for each group). Mice were sacrificed 56 days after implantation; the size of the tumor was then examined using the Xenogen IVIS imaging system. High luciferase activity was observed in the left flanks of mice that received cells transfected with the control virus, whereas significant reductions in luciferase activity (Figure 5B) and average tumor size (Figure 5C) were observed in the LV-miR-199a-3p group. The tumors were resected and processed for immunohistochemical staining; the results revealed that treatment with the LV-miR-199a-3p construct resulted in the decreased expression of RAP2a and DNMT3a in tumor tissues (Figure 5D). These results indicate that miR-199a-3p suppresses the proliferation of BCPAP cells through the targeting of RAP2a and DNMT3a *in vivo*.

## Discussion

In the present study, we describe the significant down-regulation of miR-199a-3p in PTC, both in primary PTC tissue and cell lines, and its action as a TSG during carcinogenesis. Most interestingly, we found that DNMT3a and RAP2a were target genes of miR-199a-3p, and that the methylation level of miR-199a-3p was regulated by DNMT3a, which implicated the miR-199a-3p/DNMT3a construct in a regulatory circuit controlling miR-199a-3p/DNMT3a expression. Meanwhile, RAP2a plays a key role in PTC cell migration, invasion, and growth. In clinical samples, RAP2a and DNMT3a were significantly overexpressed in PTC, and the expression of RAP2a was inversely correlated with the expression of miR-199a-3p. Our *in vivo* experiment further confirmed that miR-199a-3p suppresses cancer development in a mouse xenograft model. Therefore, our results revealed that an epigenetic change in the promoter region of miR-199a contributes to the aggressive behavior of PTC via the miR-199a-3p/DNMT3a regulatory circuit and direct targeting of RAP2a (Figure 5E).

Epigenetic modification, including DNA methylation, histone modification, acetylation and ncRNAs, is a mechanism for the regulation of gene expression (Baylin and Ohm, 2006). Recently, miR-199a was reported to be linked to some aggressive tumor types, such as gastric cancer (Ueda et al., 2010), bladder cancer (Ichimi et al., 2009), uveal melanoma (Yin et al., 2010), ovarian cancer (Iorio et al., 2007; Chen et al., 2008; Nam et al., 2008), and malignant testicular tumors (Cheung et al., 2011). MiR-199a-3p has also been reported as a tumor suppresser gene in PTC (Liu et al., 2017); however, the underlying mechanisms remain unclear. The antiproliferative and anti-invasive properties of miR-199a-3p demonstrated in this study further support the tumor suppressive role of this miRNA.

Interestingly, previous findings suggested that hypermethylation-dependent silencing of miR-199a-3p directly regulates the expression of DDR1 in ovarian cancer (Deng et al., 2017). Moreover, the enhancement of miR-199a methylation results in the down-regulation of miR-199a-3p, and miR-199a-3p inhibits aurora kinase A and attenuates xenograft tumor growth in prostate cancer (Qu et al., 2014). Loci of miR-199a were identified on two different chromosomes, Chr 1 and Chr 19. A CpG-rich region was identified upstream of the transcription start site of the miR-199a gene at both loci. Both miR-199a-3p and miR-199a-5p are derived from the same precursor RNA, but only miR-199a-3p was identified as being down-regulated in papillary thyroid carcinoma and correlated with its metastasis. The reason why only one miRNA is correlated with PTC malignancy is not clear, but it is possibly due to the differential stability of the mature miRNA molecules. Therefore, epigenetic mechanisms may act to regulate miR-199a expression in PTC. In this study, we used qRT-PCR to assay miR-199a-3p expression levels in PTC tissues and non-cancerous thyroid tissues. We found that miR-199a-3p was significantly down-regulated in PTC tissues when compared with non-cancerous thyroid tissues. We also showed that miR-199a-3p is hypermethylated in PTC tissues compared with non-cancerous thyroid tissues. Then, we used immunohistochemistry to assay the expression levels of the DNA-methyltransferases DNMT1, DNMT3a, and DNMT3b, which lead to hypermethylation, in PTC tissues and non-cancerous thyroid tissues. We found that DNMT3a, but not DNMT1 or DNMT3b, was significantly up-regulated in PTC tissues compared with non-cancerous thyroid tissues. Therefore, we further doubt that DNA hypermethylation leads to the down-regulation of miR-199a-3p expression. Next, we found that miR-199a is hypermethylated in PTC cell lines, accompanied by the increased expression of DNMT3a. Knocking down DNMT3a significantly decreased the methylation level of miR-199a; moreover, treatment of the PTC cell line with 5-aza remarkably decreased the methylation level of miR-199a. Thus, we confirmed that DNMT3a results in DNA hypermethylation, leading to the down-regulated expression of miR-199a-3p.

Subsequently, we found that decreasing the expression of miR-199a-3p caused high ectopic expression of RAP2a and DNMT3a in PTC. Bioinformatics analysis revealed a potential target site at the 3′-UTR of RAP2a and DNMT3a mRNA, which was targeted by miR-199a-3p. DNMT3a, as a crucial regulator of methylation, mediates the epigenetic silencing of TSGs and contributes to cancer progression (Chen and Chan, 2014). Previous evidence showed that DNMT3a was a target of miR-199a-3p in testicular cancer (Chen et al., 2014). Our previous study also reported that DNMT3a was a direct target of miR-204, and DNMT3a was responsible for the hypermethylation of miR-204 in the process of HA-VSMC calcification (Lin et al., 2018). In the current study, we confirmed that DNMT3a showed up-regulation in PTC tissues in comparison with normal thyroid tissues and that miR-199a-3p mimics could reduce the expression level of DNMT3a in PTC cell lines. While the suppression of miR-199a-3p significantly up-regulated DNMT3a expression levels in PTC cells, these observations suggest an epigenetic regulatory role for miR-199a-3p in PTC. Because of promoter hypermethylation, a reduction in miR-199a-3p expression induces the up-regulation of DNMT3a, which in turn enhances hypermethylation. Thus, a positive feedback loop may play a critical role in maintaining hypermethylation status and the silencing of miR-199a-3p in PTC. Moreover, we identified RAP2a as another target of miR-199a-3p. RAP2a belongs to the Ras-related small GTP-binding protein superfamily, which has been reported to affect tissue invasiveness and metastasis in many human cancers (Prabakaran et al., 2011; Wu et al., 2015). Although limited studies have suggested that RAP2a can stimulate cell growth in androgen-dependent LNCaP human prostate cancer cells (Bigler et al., 2007), and that the high expression of RAP2a has a potential relationship with advanced primary tumor status and advanced TNM stage in nasopharyngeal cancer (Lee et al., 2015), another RAP protein, RAP1, which has a structural similarity to the closely related RAP2, has been found in various human cancers and cell lines, such as pancreatic cancer (Zhang et al., 2006), prostate cancer (Bailey et al., 2009), colon cancer (Tsygankova et al., 2010), and squamous cell carcinoma of the head and neck (Chen et al., 2013). Another study found that RAP2a inhibits glioma cell migration and invasion by down-regulating p-AKT (Wang et al., 2014). To explain these diverse findings, we knocked down RAP2a in PTC cells and found that the silencing of RAP2a inhibits proliferation, migration, and invasion in these cells. Moreover, RAP2a was identified as highly expressed in PTC tissues, and much lower expression was detected in non-cancerous thyroid tissues. Furthermore, we found a negative correlation between RAP2a levels and miR-199a-3p in patient specimens. These results suggest that RAP2a might be a tumor-promoting gene in PTC, but more basic functional studies are needed to clarify the regulatory role of RAP2a in PTC progression *in vivo*.

We also used a nude mouse xenograft model to confirm that miR-199a-3p could suppress PTC cell proliferation and tumor volume *in vivo*. Immunohistochemistry staining showed that the expression of RAP2a and DMNT3a was decreased in a xenograft model with miR-199a-3p overexpression, which suggests that both RAP2a and DMNT3a were targets of miR-199a-3p, and RAP2a and DMNT3a participate in the tumor suppression effect of miR-199a-3p.

In conclusion, our data revealed that the low expression of miR-199a-3p is associated with DNA methylation of its promoter in thyroid cancer. MiR-199a-3p could target DNMT3a and RAP2a, and the miR-199a-3p/DNMT3a regulatory circuit caused the accelerated methylation of miR-199a-3p, the down-regulation of miR-199a-3p, and the up-regulation of RAP2a. Therefore, more investigations are needed to explore the potential role of miR-199a-3p and RAP2a as novel therapeutic targets of PTC.

## Data Availability Statement

The original contributions presented in the study are included in the article/Supplementary Material, further inquiries can be directed to the corresponding author.

## Ethics Statement

The studies involving human participants were reviewed and approved by the Ethics Committee of the Second Xiangya Hospital, Central South University. The patients/participants provided their written informed consent to participate in this study. The animal study was reviewed and approved by the Ethics Committee of the Second Xiangya Hospital, Central South University.

## Author Contributions

L-QY conceived and designed the manuscript. FW, XL, S-KS, FL, FX, J-YZ, BG, M-HZ, YW, and Z-HM analyzed the data. L-QY and FW wrote the manuscript. All authors contributed to the article and approved the submitted version.

## Conflict of Interest

The authors declare that the research was conducted in the absence of any commercial or financial relationships that could be construed as a potential conflict of interest.

## Supplementary Material

Supplementary Figure 1 DNMT1 and DNMT3b were detected at very low levels in normal thyroid tissues and PTC tissues. **(A)** The expression of DNMT1 was assayed by immunohistochemistry staining, and the proportion of positive cells was analyzed. **(B)** The expression of DNMT3b was assayed by immunohistochemistry staining, and the proportion of positive cells was analyzed. Bars = 200 μm.

Supplementary Table 1 Clinical characteristics of all patients and donors, related to Figure 4.

Supplementary Table 2 The primers sequences of RAP2A and DNMT3a.
