# Supplementary figures and images for: The Suppression of miR-199a-3p by Promoter Methylation Contributes to Papillary Thyroid Carcinoma Aggressiveness by Targeting RAP2a and DNMT3a
Source: Front Cell Dev Biol. 2020 Dec 7;8:594528. doi: 10.3389/fcell.2020.594528 (PMC7750465; doi:10.3389/fcell.2020.594528)

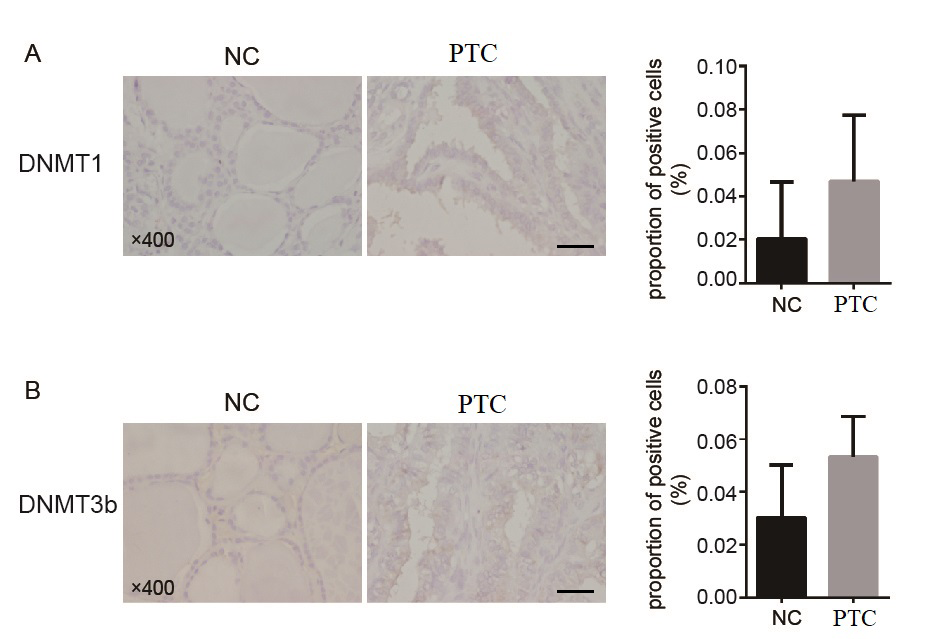

Supplement: Supplementary Figure 1 — DNMT1 and DNMT3b were detected at very low levels in normal thyroid tissues and PTC tissues. (A) The expression of DNMT1 was assayed by immunohistochemistry staining, and the proportion of positive cells was analyzed. (B) The expression of DNMT3b was assayed by immunohistochemistry staining, and the proportion of positive cells was analyzed. Bars = 200 μm. [file Image_1.tif]
