# Supplementary material for: The Suppression of miR-199a-3p by Promoter Methylation Contributes to Papillary Thyroid Carcinoma Aggressiveness by Targeting RAP2a and DNMT3a
Source: Front Cell Dev Biol. 2020 Dec 7;8:594528. doi: 10.3389/fcell.2020.594528 (PMC7750465; doi:10.3389/fcell.2020.594528)
Supplement: Supplementary Table 1 — Clinical characteristics of all patients and donors, related to Figure 4. [file Table_1.docx]

|  | PTC patients | donors |
| --- | --- | --- |
|  | n(%) | n(%) |
| Age | 43.6 ± 12.3 | 28.5 ± 15.5 |
| Female | 57 (95) | 48 (80) |
| Male | 3 (5) | 12 (20) |
| Tumor size | 18 ± 6 (mm) | - |
| Unifocal metastases | 5(8.3) | - |
| Multifocal metastases | 55(91.7) | - |

**Supplemental table 1: Clinical characteristics of all patients and donors, related to Figure 4.**
