# Supplementary material for: The Suppression of miR-199a-3p by Promoter Methylation Contributes to Papillary Thyroid Carcinoma Aggressiveness by Targeting RAP2a and DNMT3a
Source: Front Cell Dev Biol. 2020 Dec 7;8:594528. doi: 10.3389/fcell.2020.594528 (PMC7750465; doi:10.3389/fcell.2020.594528)
Supplement: Supplementary Table 2 — The primers sequences of RAP2A and DNMT3a. [file Table_2.docx]

| name | primers |
| --- | --- |
| WT RAP2A | 5' GGGTTTAAACTGCTTTGATGATTTTCTC 3' |
|  | 5' GCTCTAGACATTAACATTCATACTGGAA 3' |
| Mut RAP2A | 5' GCCATCACCTTTCTGTGGTAATAAGAGTGATATTTGC 3’ |
|  | 5' GCAAATATCACTCTTATTACCACAGAAAGGTGATGGC 3' |
| WT DNMT3a | 5' GGGTTTAAACCGCTGTTACCTCTTGTTT 3' |
|  | 5' GCTCTAGATTACTCATCTCGCTGTTT 3' |
| Mut DNMT3a | 5' AAAAGGTACTGTTAACAGCGGTACAACCCGAC 3' |
|  | 5' GTCGGGTTGTACCGCTGTTAACAGTACCTTTT 3' |

**Supplemental table 2. The primers sequences of RAP2A and DNMT3a.**
